# Childhood angular kyphosis: a plea for involvement of the pediatric neurosurgeon

**DOI:** 10.1007/s00381-017-3389-y

**Published:** 2017-03-25

**Authors:** E. Cornips, S. Koudijs, J. Vles, L. van Rhijn

**Affiliations:** 1 Department of Neurosurgery, Maastricht University Medical Center, Oxfordlaan 10, 6229 EV Maastricht, The Netherlands; 2 Department of Child Neurology, Maastricht University Medical Center, Maastricht, The Netherlands; 3 Department of Orthopedic Surgery, Maastricht University Medical Center, Maastricht, The Netherlands

**Keywords:** Case management, Child, Kyphosis, Neurosurgeon

## Abstract

**Introduction:**

Childhood angular kyphosis is rare, as most children are affected by a mixed kyphotic and scoliotic deformity. Published series involving a mix of kyphosis and kyphoscoliosis, pediatric and adult, congenital and acquired cases are almost exclusively authored by orthopedic surgeons, suggesting that (pediatric) neurosurgeons are not involved.

**Case series:**

We present five cases that illustrate the spectrum of angular kyphosis, and these were treated by a multidisciplinary team including child neurologist, orthopedic surgeon, and pediatric neurosurgeon as complementary partners.

**Discussion:**

Angular kyphosis is a cosmetic problem but above all a serious threat to the spinal cord and as such to the child’s ambulatory, sphincter, and genito-urinary functions. Spinal cord stretch over the internal kyphosis may cause pain and/or neurological deficit, often accompanied by myelomalacia or even segmental cord atrophy. Spinal cord function may be additionally affected by associated disorders such as syringomyelia or tethered cord, an orthopedic surgeon may be less familiar with. The decision when and how to proceed surgically should be made by a multidisciplinary team, including a pediatric neurosurgeon who actively participates in the operation and helps to safely achieve adequate spinal cord decompression and stabilization.

**Conclusion:**

Childhood angular kyphosis is a complex, heterogeneous disorder that should be managed by a multidisciplinary team in specialized pediatric spine centers. While every case is truly unique, the spinal cord is always at risk, especially during decompression, stabilization, and eventual correction of deformity. Pediatric neurosurgeons have an important role to play in preoperative work-up, actual operation, and follow-up.

## Introduction

Kyphosis is a sagittal plane deformity characterized by an abnormal posterior convex angulation of a segment of the spine [1]. Pure so-called angular kyphosis is quite rare in children, but may have serious consequences if left untreated [1]. Most children are affected by a mixed kyphotic and scoliotic deformity (so-called kyphoscoliosis) [1–4]. Consequently, published series involve a mix of kyphosis and kyphoscoliosis cases, pediatric and adult cases, and congenital as well as acquired cases [1–11]. The former includes cases caused by failure of formation, failure of segmentation, a mixture of both, as well as unclassifiable cases [1, 11]. The latter include cases that occur in the setting of neurofibromatosis, trauma (posttraumatic kyphosis), tuberculosis (Pott’s disease), and myelomeningocele, among others [1]. Moreover, published series are almost exclusively authored by orthopedic surgeons, suggesting that neurosurgeons in general, and pediatric neurosurgeons in particular, are not involved in caring for these children [1–4, 6, 7, 9, 10]. We present five cases that illustrate the clinical spectrum of childhood angular kyphosis, and these were treated by a multidisciplinary team including a child neurologist, an orthopedic surgeon, and a pediatric neurosurgeon as complementary partners during the decision making, surgical treatment, and follow-up process. We believe that these cases clearly illustrate the complex, heterogeneous characteristics of childhood angular kyphosis, as well as the added value of a multidisciplinary team approach.

## Case series

**Fig. 1:**
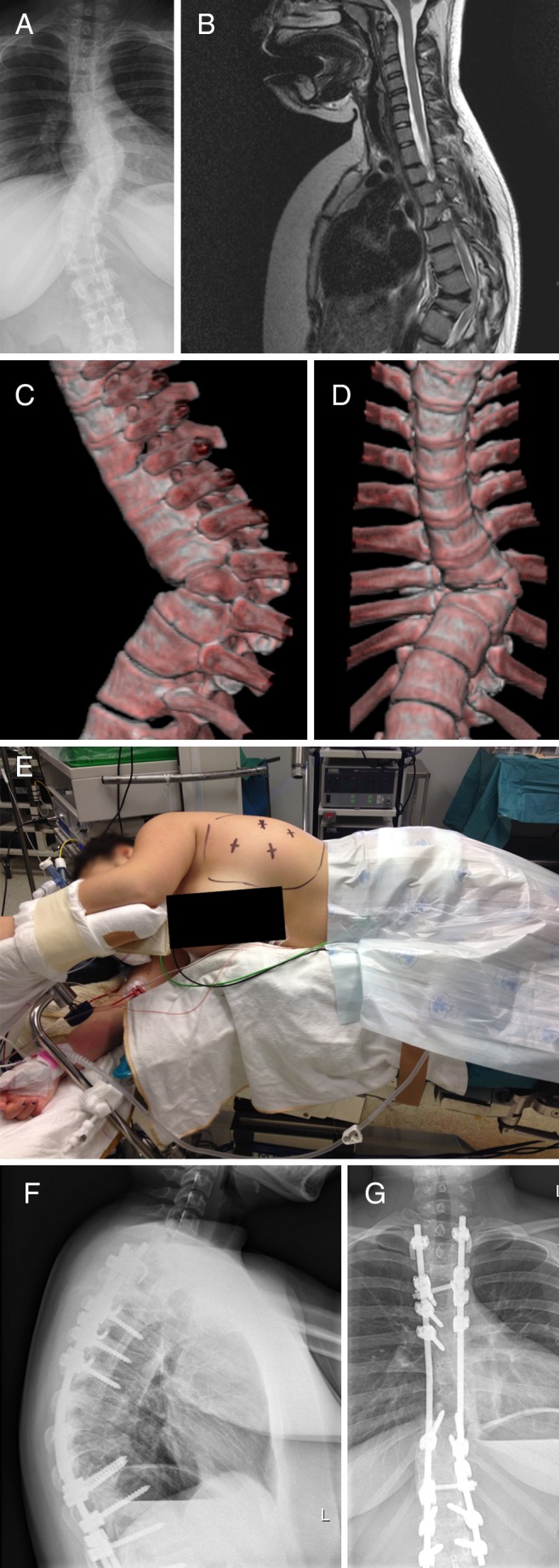
(Case 1): Girl with T8 hemivertebra, angular kyphosis, and slight scoliosis without any complaints. **a** Antero-posterior X-ray. **b** Sagittal T2-weighted MRI. **c** Lateral 3D CT reconstruction. **d** Antero-posterior 3D CT reconstruction. At age 15, the appearance of an intramedullary T2-hyperintens signal prompted prophylactic surgery, starting with an anterior thoracoscopic hemivertebrectomy and multi-level anterior release (**e**), followed by posterior redression and spondylodesis. Lateral (**f**) and antero-posterior (**g**) X-ray demonstrate partial correction of deformity

Case 1 (Fig. 1). This girl was followed several years for a stable angular kyphosis caused by a dysplastic T8 hemivertebra (vertebral body essentially absent on the left side). Besides a moderate kyphotic and slight scoliotic deformity, she had no complaints and was functioning normally. At age 15, although her deformity had not significantly progressed, she developed an intramedullary T2-hyperintens signal suggesting a developing myelopathy (myelomalacia) at the involved level. After a multidisciplinary team discussion, we advised prophylactic surgery before she would develop any symptoms or signs. We decided to start with an anterior transthoracic hemivertebrectomy from the convex (left) side. Using our experience with video-assisted thoracoscopic microdiscectomies [12, 13], we performed an anterior thoracoscopic hemivertebrectomy and release of adjacent levels with motor evoked potential (MEP) monitoring support, followed by an open posterior redression and stabilization procedure four levels above and below 10 days later. She made a swift recovery and has been doing fine with a follow-up of 3 years to date. Three-month-postoperative X-ray and MR scan demonstrated partial correction of kyphosis, adequate decompression of the spinal cord, and minimal residual myelopathy.

**Fig. 2:**
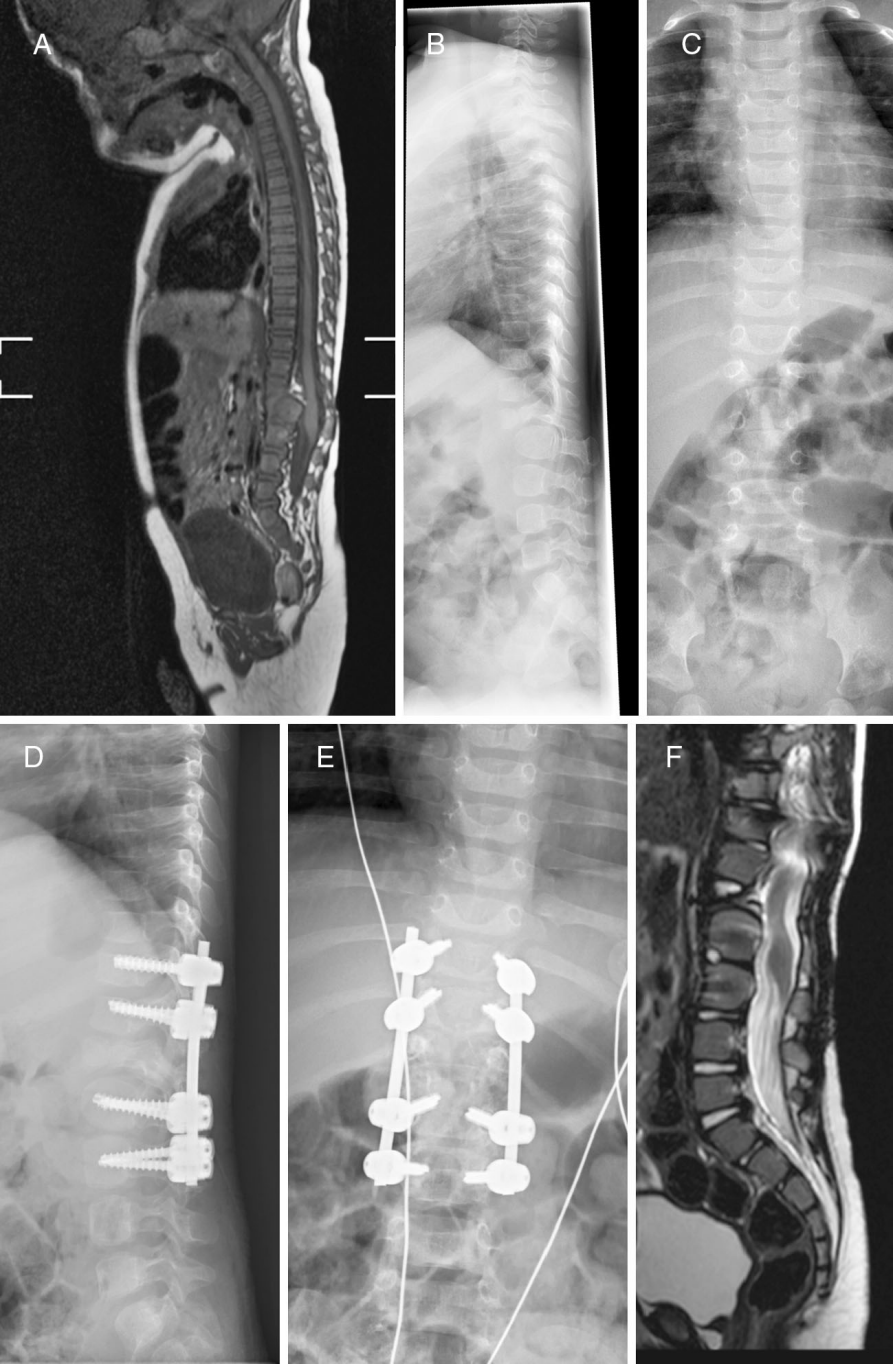
(Case 2): Boy with butterfly vertebra T12, severely hypoplastic vertebra L1, and anteroposition of T12 on L1 causing angular kyphosis with >50% antero-posterior narrowing of the spinal canal (**a**–**c**). Because the thoraco-lumbar junction was unstable and the spinal cord at risk, he was given a thoracolumbar brace until he was stabilized from T11-L3 at age one (**d**, **e**). Postoperative MR scan demonstrates improved alignation and a free lying conus (**f**)

Case 2 (Fig. 2). This boy was born with a butterfly vertebral body and dysplastic posterior elements at T12, as well as a severely hypoplastic vertebral body and posterior elements at L1. We noted an impressive anteroposition of T12 on L1, resulting angular kyphosis, and well over 50% antero-posterior narrowing of the spinal canal at this level. The thoraco-lumbar junction (still above the level of the conus) was clearly unstable as cautiously demonstrated under continuous X-ray imaging. While the spinal cord at the involved level appeared slightly hyperechogenic on diagnostic ultrasound, a T2-hyperintens signal suggesting a developing myelopathy (myelomalacia) was not confirmed on MR images. Nevertheless, we believed the spinal cord was at great risk due to repetetive movements at the thoraco-lumbar junction especially once the child was going to ambulate. His neurological and urological examinations being completely normal, he was given a thoracolumbar brace 23 h a day and carefully followed by a multidisciplinary team involving a child neurologist, pediatric neurosurgeon, and orthopedic surgeon among others. At 8 months, neurological examination was normal, while urodynamics revealed a dyssynergic pattern for which intermittent catheterization was advised. At 1 year, his spine was stabilized from T11 to L3 through a posterior approach using the Vertex® system and autologous bone supplemented by Vitoss®. He has been doing fine with normal neurological examination and a follow-up of two-and-a-half years to date. Ten-month-postoperative MR scan demonstrated improved alignation and a free lying conus.

**Fig. 3:**
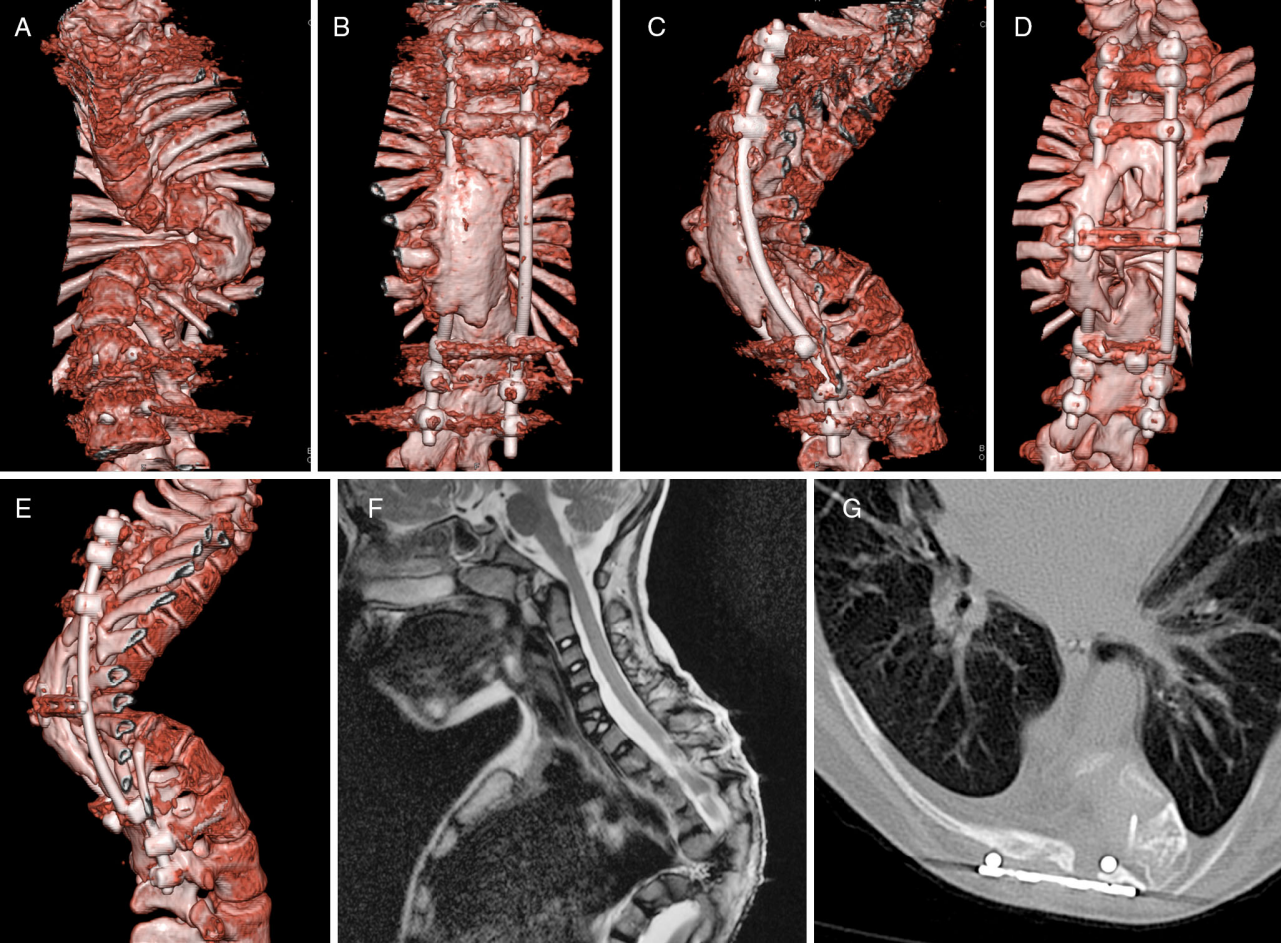
(Case 3): Girl with Conradi-Hünerman syndrome, multiple vertebral anomalies, and severe kyphoscoliotic deformity. She had a left-sided thoracotomy and posterior hemi-epiphysiodesis from T4-L1 at 17 months, and several reoperations including thoraco-lumbar wedge osteotomies and lengthening of osteosynthesis from T1-L3 (**a**–**c**). At age 10, we had to decompress a developing myelopathy at T11, removing part of the solid fusion unilaterally and adding a small transverse plate in order to protect the frail spinal cord in an essentially subcutaneous position (**d**–**g**)

Case 3 (Fig. 3). This girl was born with Conradi-Hünerman syndrome (chondrodysplasia punctata) and multiple vertebral anomalies including a 70° sinistroconvex T6-T9 scoliosis, a 45° dextroconvex T10-L2 kyphoscoliosis, and an L2-L3 anterolisthesis. While several lower thoracic vertebral bodies were severely hypoplastic on the concave side of scoliosis, MR imaging did not reveal spinal cord compression or tethering. Neurological examination at 9 months was normal except for a 2-cm longer left leg with slightly increased muscular tone. Because of the severity of the deformity, she was operated at the age of 17 months through a left-sided thoracotomy followed by a posterior approach from T4 to L1 (hemi-epiphysiodesis at the convex side using autologous bone and the CD horizon® system). In subsequent years, she was reoperated several times to lengthen the rods and to correct kyphosis (open thoraco-lumbar wedge osteotomies and posterior lengthening of osteosynthesis from T1-L3). At age 10, she presented with a slowly progressive paresis of her left leg (clinical myelopathy) and a new T2-hyperintens signal at L1-L2 (radiological myelopathy) despite a solid bony fusion. After a long discussion, both orthopedic surgeon and pediatric neurosurgeon agreed the best option was to decompress her severely compromised spinal cord (T11 vertebral level) through a posterior approach using MEP monitoring support. This involved carefully removing one of the rods and part of the bony fusion unilaterally in order to gain access, subsequently removing the compressive bony structures anterior to the spinal cord, and finally adding a small transverse plate to the construct in order to protect the frail spinal cord in its subcutaneous position at the apex of kyphosis. She recovered well after an initial decrease of strength in the left leg, and is being followed very closely.

**Fig. 4:**
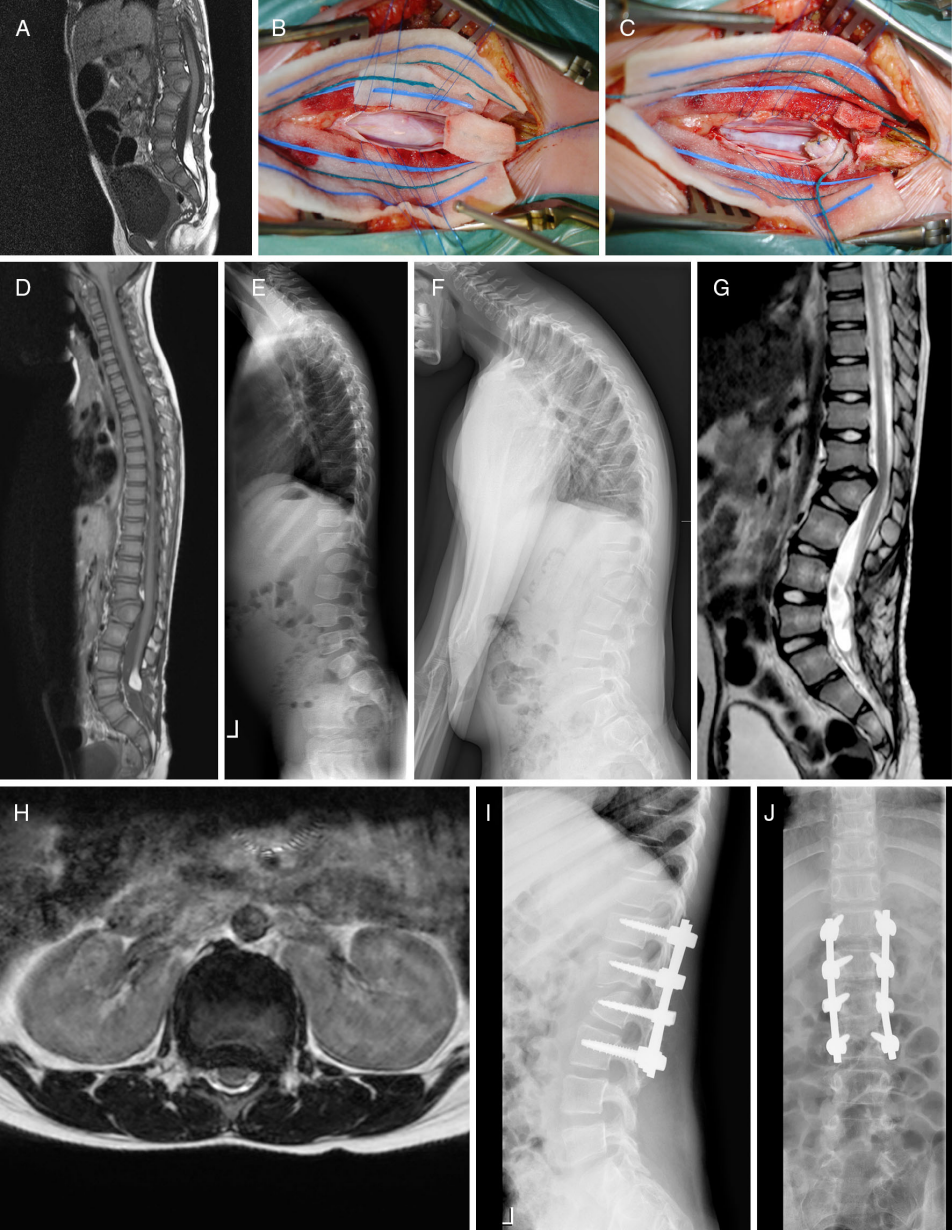
(Case 4): Girl with congenital upper lumbar kyphosis, L2 hemivertebra, and tethered cord with lipomatous component at L4-L5 (**a**). Images before (**b**) and after (**c**–**e**) lipoma debulking at age 2, re-untethering at age 7, and before (**f**–**h**) and after (**i**, **j**) T12-L3 spondylodesis at age 9. Of note, **f** is a flexion image, whereas **g**, **h** illustrate broad-based spinal cord compression at the apex of angular kyphosis at L1-L2

Case 4 (Fig.4). This girl was born with a kyphotic upper lumbar spine, lower lumbar skin fold, and normal neurological examination. Imaging studies revealed an L2 hemivertebra, angular kyphosis of 39°, and rather narrow spinal canal with normal looking spinal cord at this level. More caudally, we noted a tethered cord with lipomatous component up to the L4-L5 disc interspace, as well as discrete hydromyelia at T11-L1. She was given a corset as soon as she started to ambulate, and while angular kyphosis did not progress, she was finally scheduled for an elective untethering procedure when she was 2 years old. At age 4, she was doing fine, and bracing was discontinued. At age 7, she complained of fatigue and intermittent pain in her right leg, with minimal urine loss during Valsalva’s and at night. The orthopedic surgeons wondered whether these were symptoms of spinal cord compression at L2; however, spinal cord signal and dimensions were completely normal at that level, and symptomatic retethering was the more likely diagnosis according to the child neurologist and pediatric neurosurgeon. Therefore, prior to an eventual stabilization of kyphosis, we performed a re-untethering with additional debulking of the lipoma. Postoperatively, complaints in the right leg disappeared, but urodynamics were slightly worse pointing towards a neurogenic bladder for which she was given Vesicare®. At age 9, in order to protect the spinal cord from repetitive straining over an unchanged angular kyphosis, she was finally stabilized with a posterior fusion from T12-L3. She has been doing fine with a follow-up of nearly 2 years to date.

**Fig. 5:**
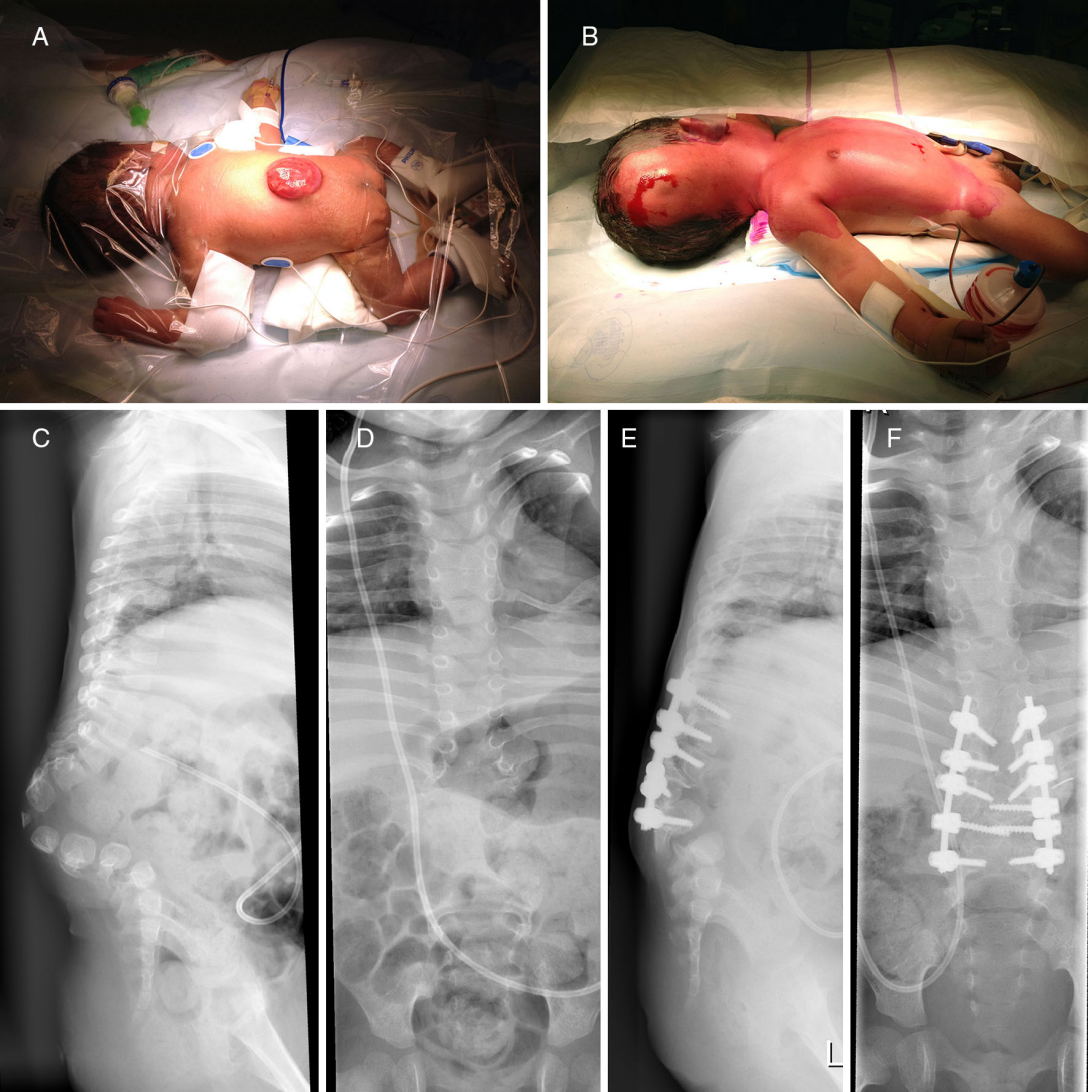
(Case 5): Newborn with large thoraco-lumbar myelomeningocele before closure (**a**) and ventriculo-peritoneal shunt placement (**b**). Severe lumbar kyphosis (**c**, **d**) was corrected at age two with an L1-L2 vertebral body resection followed by a T9-L5 spondylodesis (**e**, **f**)

Case 5 (Fig.5). This unfortunate boy was born paraplegic because of a large thoraco-lumbar myelomeningocele, which was closed on day 1 using a Z-plasty, followed by ventriculo-peritoneal shunt placement. Besides an associated Chiari II malformation, we noted lumbar kyhosis, compensatory hyperlordosis at the thoraco-lumbar junction, and segmentation anomalies (synchondrosis) as well as a syrinx at T2-T4. During follow-up by our multidisciplinary spinal dysraphism team, he developed a small decubitus at the apex of kyphosis treated conservatively. At age 2, kyphosis was corrected for obvious reasons (cosmesis, avoiding repeat decubitus, while improving sitting balance) with an L1-L2 vertebral body resection followed by a T9-L5 spondylodesis using the Vertex® system and autologous bone. While the pediatric neurosurgeon assisted during opening (avoiding inadvertent trauma to the thecal sac), a plastic surgeon assisted during closure (ensuring adequate covering of the implant). Regarding the circumstances, he has been doing fine with a follow-up of two-and-a-half years to date.

## Discussion

### Childhood angular kyphosis

Angular kyphosis whether isolated or part of a more complex kyphoscoliotic deformity of the thoracic or thoraco-lumbar spine is a serious threat to the spinal cord and as such to the child’s ambulatory, sphincter, and genito-urinary functions [1, 3, 4]. Progressive deformity may be the result of a congenital failure of formation and/or segmentation of one or more vertebrae [1, 11]. On the other hand, deformity may develop despite normal initial vertebral development following spinal trauma or infection [1]. Finally, deformity may develop in children affected by neurofibromatosis or a myelomeningocele [1]. Angular kyphosis creates a biomechanical environment promoting progression, because an anterior shift in the individual’s center of gravity results in more flexion bending moments around the apex of kyphosis [2]. Besides an obvious cosmetic problem, spinal cord stretching over the internal kyphosis may cause pain and/or neurological deficit, the latter often accompanied by myelomalacia or even segmental cord atrophy. Indeed, these children will often manifest a reduced antero-posterior spinal cord diameter, and occasionally their spinal cord may hardly be noticeable at all at the apex of kyphosis [4]. Neurological deficits are more common with failure of formation causing a sharp angular kyphosis, or with kyphosis in the upper thoracic area known as a watershed area (poor collateral circulation) for blood supply to the spinal cord. They may occur early but more frequently during preadolescent growth spurt parallelled by a rapid increase in the untreated kyphotic deformity [1]. They may occur spontaneously or after minor trauma [1].

### Other factors affecting spinal cord and spinal deformity

In the context of angular kyphosis, the spinal cord may be affected by other factors including posttraumatic arachnoiditis, syringomyelia, or a tethering mechanism, all of which may aggravate and accelerate deformity through secondary dysbalance of the erector spinae muscles. Spinal cord tethering may occur in the setting of any associated open or closed dysraphic disorder such as tight filum, lipoma, lipomeningomyelocele, meningomyelocele, or split cord malformation. Closed spinal dysraphism and especially tight filum or even split cord malformation may be overlooked by less experienced eyes even on full spine MR imaging series. Diagnosis may require additional MR sequences such as coronal images or consecutive thin-sliced axial T2-weighted images at the suspect level. Moreover, diagnosis requires a certain level of suspicion and familiarity with these essentially neurological disorders well known to child neurologist and pediatric neurosurgeon but maybe less to the orthopedic surgeon. It is therefore essential, and in our hospital part of the protocol regarding children with spinal deformity (congenital or acquired), they are evaluated at least once by a child neurologist, preferably before they have (full spine) MR imaging, which may then be ordered directly by the child neurologist. Subsequently, any suspect case is discussed with the pediatric neurosurgeon, and additional (e.g., urological) evaluation is ordered whenever deemed necessary (e.g., in dysraphic cases).

### Multidisciplinary team approach

Once the correct etiological diagnosis established with or without an eventual neurological substrate, we strongly believe the decision when and how to proceed surgically should be made by a multidisciplinary team including a child neurologist, pediatric neurosurgeon, and orthopedic surgeon. In case 1 with a T8 hemivertebra and non-significantly progressive angular kyphosis, a T2-hyperintens signal appearing on follow-up MRI prompted prophylactic surgery. In case 2 with a severely hypoplastic L1 vertebra, the thoraco-lumbar junction was unstable, and the spinal cord is at great risk especially once the child was going to ambulate. His neurological and urological examination being completely normal, he was carefully followed until age 1, allowing individual vertebrae to grow stronger before being stabilized. In case 3 with chondrodysplasia punctata and previous surgeries to stabilize a severe kyphoscoliotic deformity, progressive paresis of the left leg corresponded with a new T2-hyperintens signal at L1-L2. Indeed, the spinal canal was very narrow, and the spinal cord severely compromised at the apex of kyphosis despite solid fusion. Part of the fusion was removed unilaterally to decompress the frail spinal cord, which was subsequently protected with a small transverse plate. In case 4 with an L2 hemivertebra and tethered cord with lipomatous component, we performed elective untethering at age 2 and re-untethering at age 7 as she developed symptoms and signs that suggested retethering rather than mechanical cord compression at L2. Postoperatively, her complaints disappeared, allowing to postpone posterior fusion until age 9. In case 5 with a thoraco-lumbar myelomeningocele, kyphosis was corrected at age 2 by a team involving a pediatric neurosurgeon (opening), orthopedic surgeon (stabilization), and plastic surgeon (closure).

These five cases illustrate the necessity of a multidisciplinary team approach at the outpatient clinic, in the OR theater, and during follow-up until the child has reached adulthood. At the outpatient clinic, the child neurologist carefully monitors the child for symptoms and signs of spinal cord compression or retethering; the orthopedic surgeon is looking for eventual curve progression, and the pediatric neurosurgeon is screening different neuroimaging studies for subtle changes that might suggest retethering, a developing myelopathy, or a progressive hydromyelia. In the OR theatre, the pediatric neurosurgeon has specific knowledge and skills to interpret information shared by the neurophysiologist (case 1, 3) and to safely decompress the spinal cord. The orthopedic surgeon has specific knowledge and skills to decide when and how to fuse these immature spines, taking into account eventual additional surgery in the future, while minimizing spinal growth compromise in the very young. Of note, in order to promote solid fusion while avoiding long segment fusion, extra bone and in selected cases bone substitutes are added in between the involved vertebral bodies and over the postero-lateral elements.

### Individualized treatment and the role of the pediatric neurosurgeon

These five cases illustrate that every child with angular kyphosis or kyphoscoliosis is truly unique with regard to its clinical and radiological presentation [1, 3, 4]. Therefore, treatment should be highly individualized and thoroughly discussed with all team members. Surgical treatment is technically demanding and potentially dangerous in terms of neurological outcome for several reasons: the spinal cord is already “sick” and may be additionally damaged during decompression, it may be distorted or it may impinge at the edges of decompression during correction, the feeding vessels may be destroyed, and profuse blood loss or even embolization may occur [3, 4]. In this context, the observation that papers regarding childhood angular kyphosis or kyphoscoliosis are published almost exclusively by orthopedic surgeons in journals not dedicated to the pediatric population suggests that pediatric neurosurgeons should be more involved [1–4, 6, 7, 9, 10]. Indeed, they should have an important role interpreting every individual’s clinical and radiological presentation, counseling the parents, deciding whether spinal cord or spinal column should be surgically addressed first, and helping the orthopedic surgeon to safely achieve adequate spinal cord decompression prior to spinal stabilization.

### Value and challenges of the learning curve

Caring for children with angular kyphosis, as for those with other complex spinal problems, implies a steep learning curve for everyone involved. All disciplines mentioned before (child neurologist, pediatric neurosurgeon, orthopedic surgeon) should be available and actively involved in the entire process. As their adult colleagues, they may learn a lot from each other. Given their specific background, their initial approach to the problem may appear quite different. However, in order to provide the best possible care for every unique case, a well-balanced view considering different treatment strategies is indispensable. Additionally, dedicated pediatric anesthesiologists are a prerequisite to ensure the child’s safety during surgery, to enable reliable intraoperative neuromonitoring (which also requires an experienced neurophysiology team), and to provide adequate pain control postoperatively in close cooperation with the pediatric intensive care team. Importantly, there should be enough case load to achieve and maintain diagnostic and surgical competence as a team, so as to avoid unnecessary operations, reoperations, and to prevent complications in these children who will often face multiple spinal surgeries before they reach adulthood. In the context of contemporary healthcare reform programs and economics, this clearly implies centralization in relatively large, specialized, pediatric spine centers.

## Conclusion

Childhood angular kyphosis is a complex, heterogeneous disorder that should be managed by a multidisciplinary team in specialized pediatric spine centers. While every case is truly unique, the spinal cord is always at risk, especially during decompression, stabilization, and eventual correction of deformity. Pediatric neurosurgeons have an important role to play in preoperative work-up, actual operation, and follow-up.

## Abbreviations

CAK: Congenital angular kyphosis
CT: Computed tomography
L: Lumbar
MEP: Motor evoked potential
MR: Magnetic resonance
MRI: Magnetic resonance imaging
T: Thoracic
